# Social economic factors and malaria transmission in Lower Moshi, Northern Tanzania

**DOI:** 10.1186/1756-3305-5-129

**Published:** 2012-06-28

**Authors:** Asanterabi Lowassa, Humphrey D Mazigo, Aneth M Mahande, Beda J Mwang’onde, Shandala Msangi, Michael J Mahande, Epiphania E Kimaro, Eliapenda Elisante, Eliningaya J Kweka

**Affiliations:** 1 Tanzania Wildlife Research Institute, P.O. Box 661, Arusha, Tanzania; 2 Department of Medical Parasitology and Entomology, Catholic University of Health and Allied Sciences, P.O. Box 1464, Mwanza, Tanzania; 3 Tropical Pesticides Research Institute, Division of Livestock and Human Disease Vector Control, Mosquito Section, P.O. Box 3024, Arusha, Tanzania; 4 KCM College of Tumaini University, P.O. Box 2240, Moshi, Tanzania; 5 Department of Physical Sciences, Faculty of Science, Sokoine University of Agriculture, P. O. Box 3038, Chuo Kikuu - Morogoro, Tanzania

## Abstract

**Background:**

For many years social economic status has been used as an indicator to characterize malaria treatment seeking behaviors of communities and their adherence to malaria control programs. The present study was therefore conducted to assess the influence of household social economic status, knowledge, attitude and practice on treatment seeking behaviors, distance to health facilities and vector control measures in the Lower Moshi area, northern Tanzania.

**Methods:**

A cross-sectional household survey was carried out, a quantitative method was used to collect information from the households, and the household socio-economic status was estimated by employing a household asset-based approach. The structured questionnaire also collected information on malaria knowledge, attitudes and treatment seeking behaviors.

**Results:**

A total of 197 (68.8% were female) household heads were interviewed**.** Distance to the health centers influenced malaria treatment seeking behaviors especially for children (*P* = 0.001) and the number of visits to the health facilities made by the household members (*P* = 0.001). The head of the households' level of education had an influence on bed-net retreatment (*P* < 0.001) and acceptability of larval control programmes (*P* <0.001). Similarly, a significant association was observed between bed-net retreatment, larval control and occupation of the head of the household .

**Conclusion:**

Distance to the health centre influenced malaria treatment seeking behaviors, and the number of visits made by the household members. In addition, the education level of the household heads played a role in understanding and in the selection of malaria interventions for the households. Increasing the number of health facilities close to rural areas will improve malaria treatment seeking behavior, case management and hence reduce malaria-associated morbidities, especially in high risk groups.

## Background

In Sub-Saharan Africa malaria remains a major cause of morbidity and mortality compared to any other region of the world, it is responsible for about 515 million clinical cases and 1.7 million deaths annually [1,2]. The Sub-Saharan Africa region carries approximately 90% of the global malaria burden [2]. About 95% of the Tanzanian population are at risk of malaria and live in areas characterized by stable malaria transmission [3]. In these areas, about 17–20 million clinical episodes of malaria are reported each year and almost 80,000 deaths are attributed to malaria every year [3,4]. In rural areas malaria contributes to approximately 40% of all outpatient visits; with children under five and pregnant women contributing the highest proportions [5-7]. Currently, there is evidence that malaria transmission is declining in many endemic areas of Sub-Saharan Africa [3,8-10]. The recent statistics in Tanzania indicate that mortalities due to malaria have decreased from 100,000 [4] to 80,000 deaths [3]. Lower Moshi is considered to be a holo-endemic area for malaria transmission [11,12]. Data from health facilities in the area indicate that malaria, upper respiratory infections, soil transmitted helminthes, intestinal protozoa and human immunodeficiency virus infections are the most common health problems [13-16]. Similar to the national malaria control strategies, the use of bed nets and treatment of active malaria cases with a dose of effective anti-malarials are the main strategies used in the area against malaria [14,17,18]. The wide distribution of bed nets through subsidized prices, popularly referred to as “hati punguzo”, to pregnant women and children under five years old has increased the ownership and protection of these vulnerable groups against malaria [19]. In addition, several intervention trials against malaria that have been conducted in the area have increased the level of bed net ownership and coverage compared to other rural areas in the country [20,21]. Although control efforts have been stepped up in the area, malaria transmission continues to be a public health problem. Findings from previous studies have reported that the majority of individuals were aware of malaria transmission and they used various interventions such as plant repellants to reduce indoor vector density [22]. In addition, the previous studies have reported that the majority of inhabitants could afford to buy treated/non-treated bed nets from local markets [20]. The results from a household-based survey, carried out in Lower Moshi rural communities are reported here. The study focused on understanding how the household social economic status, knowledge, attitude and practice influence malaria treatment seeking behaviors and vector control measures in the Lower Moshi area, northern Tanzania.

## Methods

### Study area

**Figure 1:**
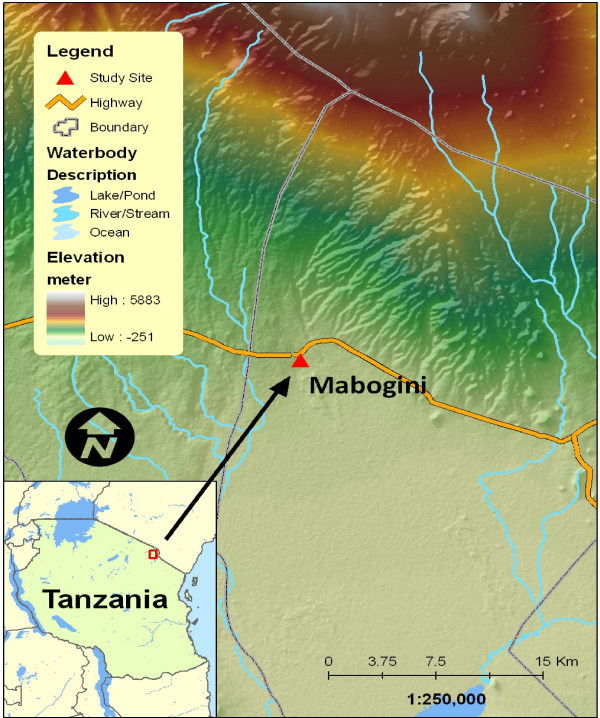
The map of Tanzania showing the area where study was conducted.

Lower Moshi is located on the southern foothills of Mount Kilimanjaro (321’S, 3721’E) (Figure 1). This area generally has an elevation ranging between 700 to 800 m above sea level. Malaria transmission occurs throughout the year with low parasitaemia [14,17,18] and low entomological inoculation rate [12,23]. The main malaria vectors in this area are *An. arabiensis* and *An. funestus*[12,23-25]. On the land surfaces, several water streams cross the area and form the irrigation channels for paddy (Oryza sativa) irrigation. The rice irrigation schemes have structured and unstructured canal networks; covering an area of about 1,100 hectares. During the rainy season, temporary pools that serve as malaria vector breeding sites are formed. Their persistence beyond the rains contributes to further malaria transmission. The area has two rainy seasons, the long rains which run from March to May and short rainy season from November to December. The average annual rainfall is 900 mm per year. In addition to paddy cultivation, the inhabitants are also involved in cultivating vegetables, maize (*Zea mays)*, peas (*Pisum sativum)* and beans (*Pisum sativum*). Inhabitants also keep domestic animals such as cattle, goats, sheep and poultry.

### Study design

This was an analytical cross-sectional survey, conducted with an objective of determining malaria vectors and larval control knowledge, use and ownership of malaria intervention tools and the wealth of the head of the households. In addition, the study assessed passive malaria cases reported at the health facility within the village and various factors that were associated with the use of malaria intervention tools. The Tropical Pesticides Research Institute distributed bed nets in Lower Moshi for evaluation in 2005 [21]. All selected head of households in this study were involved in the previous bed net distribution and evaluation programme. These nets were not part of free net programmes, which were intended for malaria control for pregnant women and children [3,26]. During community surveys a total of 1976 households were visited to characterize their willingness to participate in malaria vector control through a community participatory approach. Thereafter, ten percent (197) of these households were randomly selected to participate in the present study. The head of the household was defined as the person who was perceived by household members to be the primary decision maker in the family; and the household was defined as individuals living together and taking meals from a common cooking facility [27]. In the absence of a household head, a responsible adult above 18 years who was appointed by the family was interviewed.

### Data collection

Data were collected using a structured questionnaire from randomly selected household heads. The data collected included knowledge, attitude and practice (KAP) regarding malaria and integrated malaria control management awareness. The questionnaires also collected information on the distance and cost incurred in visiting the health facilities for malaria treatment. Demographic information of the household heads was also collected. Information on property ownership (radio, bicycle, animals, land, and house light source) was also collected for the purpose of assessing the contribution of wealth on malaria awareness and control. The bed net coverage, treatment and physical status (with or without holes) of bed nets was assessed and related to malaria control knowledge. Data of malaria positive confirmed cases were collected from medical records of the health centre, with the name of patient’s village and the period of study when it was conducted. In addition, information on common diseases co-infecting children with malaria in the study area were collected from the health facility records in the study area. The distance from household to nearby health facilities was a factor taken into consideration to characterize community health-seeking behavior.

### Data analysis

Double data entry was done using Microsoft Access for validation purpose before analysis. Statistical analysis was done using PWAS statistics 18 (Version 18.0 for Windows, SPSS Inc., Chicago, IL). The effect of distance to the health centre was calculated using a Poisson regression. One way Analysis of Variance (ANOVA) to determine the difference of means between education levels versus ITN ownership; education versus ITN re-treatment, family income and treatment costs coverage. The factor was considered significant when the *P*-value was below 0.05.

### Ethical consideration

Ethical approval was obtained from The Tropical Pesticides Research Institute ethics committee, Arusha Tanzania prior the commencement of this study. This study was conducted within the main study of the evaluation of bed nets in this area. Permission to conduct the study was also sought from the district authority of the respective study area and from individuals before entering their homes. Meetings were held in each village and the objectives of the study were clearly described to villagers in Kiswahili language, the language inhabitants understood very well. Participants were also informed that participation in the study was on a voluntary basis. Before recruitment into the study, participants were provided with written informed consent; and confidentiality of the all the information collected from households was maintained.

## Results

A total of 197 heads of household were interviewed and their median age was 35.8 (24 to 84) years. The majority of the household heads were Christian (53.1%) and over two thirds of them were females, 68.8% (136/197). Of all the households, 94.39% (185/197) had primary school education, 8 (4.08%) had secondary school education and the remaining 1.53% were illiterate. All 197 (100%) household heads knew about malaria vectors and their role in transmission of malaria. In addition, the household heads had skills and were aware of techniques used to reduce vector-human contact and 80% of them reported a treatment seeking behaviour for malaria.

**Figure 2:**
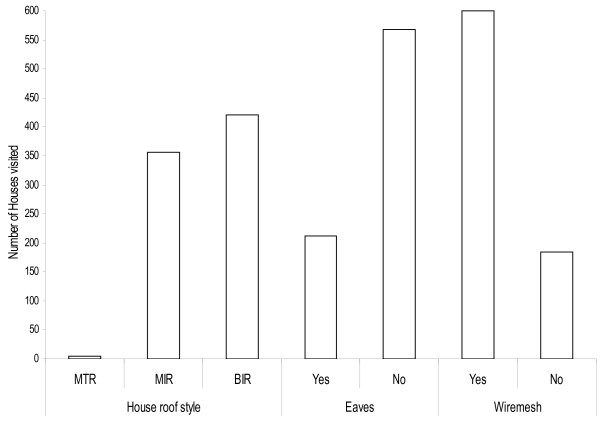
**The house characte** ***ristics and*** **improvements in area of lower Moshi irrigation scheme, House styles; Mud w** ***all and th*** **atches roof (MTR), Mud wall and Iron roof (MIR), Brick wall and Iron roof (BIR) and presence or Absence of eaves and wire mesh.**

On the use of malaria prevention tools, 195 (98.9%; 95% CI: 97.01-98.9) of the household heads reported owning and using bed nets for controlling malaria transmission. Among those who reported owning bed nets, 95.4% (188/197; 95% CI: 94.9 -95.9) owned conventionally treated bed nets, and 3.6% (7/197;; 95% CI: 2.9-3.9) owned untreated bed nets. A small proportion, 1.0% (2/197; 95% CI: 0.85 -1.2) had no bed nets. None of the studied households reported to own a long lasting insecticide-treated bed net. The wealth of the household heads was also assessed based on equipment owned by the household. The mean household size was 5.3 individuals per household. The average amount of money spent on malaria prevention tools are shown in Table 1. The mean annual earning of the household heads ranged from 720,000 to 1,440,000 Tanzanian Shillings (516.7 to 1,033.4 USD). In addition, 45.2% (89/197; 95% CI: 44.3 – 46.9) of the household heads kept domestic animals in their household. The majority of them kept cattle, goats, sheep and poultry. All of the interviewed household heads were residents of Mabogini for more than 15 years and the majority of them 99.5% (196/197; 95% CI: 98.9 – 99.7) knew the areas where malaria vector larvae are found and the time when larvae productivity was high. In fact, the household heads knew that if their houses were close to the mosquito breeding habitats it increased the risk of exposure to mosquito bites and subsequently malaria transmission**.** The types of houses owned by the household heads varied in design and quality (Figure 2), A total of 811 malaria confirmed cases were found in the health center; of whom 70.28% (570/811) were children. The distance to the health centre (regarded as a factor for health facility accessibility and cost to be met by patients) significantly influenced malaria treatment seeking behavior among participants (Table 2). Distance to the health centre influenced treatment seeking behaviour for malaria especially for children (OR = 2.4; 95% CI: 1.6 – 2.7, *P* = 0.001). No significant association was observed between education level and ownership of bed nets (F = 1.813, DF = 195, *P* = 0.943), mosquito net treatments (F = 0.258, DF = 195, *P* = 0.773), malaria treatment costs (F = 3.278, DF = 195, *P* = 0.400) and family income (F =0.869, DF = 193, *P* = 0.421). Interestingly, the education level of the household heads was strongly associated with awareness of malaria vector larval control carried out by different authorities (F =63.343, DF = 195, *P* > 0.001) and bed-net re-treatments (F = 163.362, DF = 193, *P* > 0.001).

**Table 1:** The response frequency and cost for mosquito human contact protection outside bed net at household level in lower Moshi per month

**Preventive measure**	**Response of household heads**	**Cost(USD) /month**
Aerosol spray	12 (6.09%)	12.17
Mosquito coils	99 (50.25%)	7.57
Synthetic repellents	2 (1.02%)	16.45
Use smoke	143 (72.59%)	0
Plant base repellents	164 (83.2	0

**Note**: 1 USD was equivalent to 1680 TShs.

**Table 2:** Health care centre visiting rates among the residents interviewed in lower Moshi living at a distance of 0 to 6.0 Km from the health centre

**Distance (In kilometer)**	**Number of patients who attended the clinic (by distance)**	**Rate of clinic visits by distance lived from clinic/person/year (95%CI)**	**Rate ratio of clinic visit by distance lived from clinic (95%CI)**
0.0-1.0	224	0.28 (0.24, 0.33)	0.52 (0.37, 0.7)
1.01-2.0	112	0.14 (0.11, 0.18)	0.26 (0.22, 0.32)
2.01-3.0	24	0.03 (0.02,0.05)	0.05 (0.03, 0.08)
3.01-4.0	16	0.02 (0.01,0.03)	0.04 (0.03,0.05)
4.01-5.0	396	0.05 (0.05, 0.07)	0.09 (0.07, 0.12)
5.01-6.0	12	0.02 (0.01, 0.03)	0.04 (0.02, 0.06)
**Overall**	DF = 195	F = 1.813	*P* = 0.049

**Figure 3:**
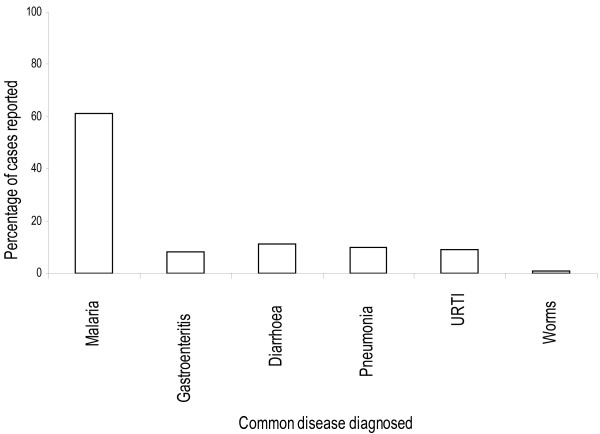
The commonly diagnosed diseases at Lower Moshi health facility for children under five years old.

In addition, household head income generating activities showed a significant association with bed-net retreatments (F = 332.227, DF = 191, *P* > 0.001). This was not true for bed net ownership (F =1.871, DF = 193, *P* = 0. 116) and food expenditure of household members (F = 1.578, DF = 193, *P* = 0.209). Based on the health reports from the health centre where the majority of the household heads and other community members get their health services, indicates that besides malaria, the area is endemic for other tropical diseases, and co-infections were found to exist (Figure 3).

## Discussion

These findings suggest that distance to the health facility from the household influenced malaria treatment seeking behaviour of most of the household heads. The main groups from the surveyed households, who were negatively affected by the observed distance, were children under five. This observation was similar to reports from previous similar studies [28,29]. Reports from other tropical diseases such as TB and HIV indicated that distance to the health centers were an impending factor for health seeking behaviour and treatments [19,28]. From this observation one can speculate that the majority of the household members whose houses are close to the health facilities will have timely and more visits to the health facility as compared to those whose households are far away from the health facility. The further the distance from the health facilities, the higher the cost an individual will incur to visit the health facilities. Despite the distance to the health facilities and the costs, household heads were willing to incur the transport costs for the sick children rather than the adults [29]. In addition, free health services offered to children under five years old, could have contributed to increased visits observed in this age group. To improve malaria treatment seeking behaviors in malaria endemic communities, locating health facilities close to the community or training community health workers may improve malaria treatment seeking behaviors, which could result in early malaria case management and hence reduction of mortality rates associated with malaria in risk groups.

Despite the fact that household income was reasonable as compared to many rural villages [30-32]**,** the annual income had no influence on expenditure for malaria treatment and control**.** This observation is similar to that of previous studies in malaria endemic countries [26,33]. However, this contrasts with findings of other similar studies in malaria endemic areas [26,33]. Probably, the free bed net distribution policy for children and pregnant women has resulted in money spent on bed nets at the household level to be low [3,4,26,34] Bed nets are one of the most recognized methods of personal protection against mosquitoes, and many studies have reported the benefits of bed nets; either insecticide treated or untreated [35,36]. In the study area, like many other areas in the country [20], use of conventional insecticide-treated bed nets was the most common protection tool for malaria. Despite the majority of households reporting to own treated bed nets, a significant number did not re-treat their bed nets at the recommended time. Thus, most of the bed nets become less protective against mosquitoes and increase the risk of malaria transmission. Net washing is an important determinant of the effectiveness of ITNs [37], and targeted efforts have to be made to sensitize the community on washing their nets after a specified period of time. A small proportion of households were observed to have untreated bed nets and others had no bed nets. Of the households visited, none owned long lasting insecticide-treated bed nets. The observation was similar to previous findings in four other villages in rural Tanzania where the bed net coverage was low and associated with high rates of malaria transmission [20].

The main malaria vectors found in the area are, *An. arabiensis* and more currently *An. gambiae* s.s has also been recorded [12,28,38]. The former species is known to be zoophilic (feeds on mammals such as cattle) and anthropophilic depending on the type of host available and geographical locations [39,40]. The presence of cattle in the households may attract additional mosquitoes to the household vicinity, resulting in zoopotentiation. Entomological studies in the same area have demonstrated that *An. arabiensis* had higher preference to animal baits rather than human being baits and odour-baited traps [25]. Conversely, use of cow urine showed similar results on behavioral response of *An. arabiensis*[41,42]. For the households without animals in their compounds, human beings are most likely to be the blood meal source than their counterparts with cattle herds [43]. From these observations, the use of ITNs or LLITNs for protection against anthropophilic mosquito species is recommended. For the zoophilic species (which bites outdoors), use of pyrethroid insecticides sprayed on animals for the households with animals, may serve to reduce mosquito density in their vicinities. Use of topical repellants is also recommended for households with or without animals in their vicinity. In addition, the paddy irrigation scheme in the area is another potential risk factor for malaria transmission as it provides breeding sites for malaria vectors throughout the year [24,38,44-48]. Thus, more integrated efforts against malaria are highly required in the area. Individuals with higher education were more health conscious and more likely to adhere to public health education as compared to illiterate people. In our study, a high education level was significantly associated with bed-net retreatment among the study participants. These findings are supported by the findings of other studies in different epidemiological settings where malaria is endemic [33,49-51]. Similarly, in the same study population, education level was associated with good awareness of the larval control strategies for reduction of malaria transmission. Surprisingly, there was no relationship between the education level of the household heads and ownership of bed nets. Our observation was contrary to previous observations where the household head’s education level was reported to be significantly associated with bed net ownership and re-treatments [33,35].

Similar to other community studies, individuals with a higher income have a positive response to public health intervention for many tropical diseases, including malaria [33]. Despite having a reasonable income to support the family members in buying ITNs and insecticides for bed-net re-treatment from the retail markets, the majority of the household heads did not adhere to utility and re-treatment of bed nets. Similar findings were reported in Western Kenya [52]. Furthermore, no association was observed between the level of bed net ownership and the household income. The distribution of subsidized ITNs among pregnant women and children under five through the “Hati Punguzo” programme and the ongoing mass distribution of free ITNs from the government have increased the coverage and ownership in the community [9,20]. However, despite these individuals having a reasonable income, the majority of the household heads did not adhere to bed-net usage or bed-net retreatment. The favorable environment for vector survival and high prevalence of tropical diseases in Sub-Saharan Africa, means that co-infections of multiple parasitic infections in an area or in a single host are common [52,53]. The multiple parasitic infections are associated with considerable morbidities in co-infected individuals [53]. In fact, co-infections of malaria and other diseases such as helminth infections tend to potentiate the effects of each other in a co-infected individual [53-55]. In addition to malaria, other diseases found prevalent amongst children in the study area were pneumonia, diarrhea, upper respiratory tract infection (URTI), gastroenteritis and intestinal helminths. Because of high prevalence of these infections, co-infections are possible. The cost spent on personal protection measures when outside the bed net at household level might have been another factor for persistence of malaria transmission in the study area, as few are protected by other means outside the bed nets. The house improvements in this study area should target the blocking of eaves, and the use of window screens among the residents, which have been reported to have great impact in other areas for reducing indoor malaria transmission [52,56-59]. Environmental management for larval control has played a major role in malaria vector reduction and elimination in different areas where the technique was deployed [59-65]; hence it has to be considered in the present study area for the larval habitat control in irrigation schemes and in other habitat types, since community members are knowledgeable about mosquito habitats. Currently, there is loss of efficacy in pyrethroids used in ITN and IRS programs against malaria vectors [66,67]. In this study area, most malaria vectors have been reported to be resistant against the pyrethroids used in public health [11,68]. Understanding continuous malaria transmission in this area remains a major factor. Malaria vector control tools have been implemented for a while in this community and seemed to lose efficacy in other parts of malaria endemic regions [66,67]. The commonly reported factor from the community is the lack of knowledge on loss of efficacy of the conventionally treated bed nets after a given period of time and numbers of washes, hence allowing mosquito feeding success and subsequently disease transmission. The findings from the current study suggest that community members in the study area can afford to buy LLITNs but seem to have lacked motivation in the importance of using LLITNs to substitute ITNs, which are not regularly re-treated as described by public health officials for best protection outcomes. Education and occupation had an influence in vector control tools, therefore, knowledge and utility might have more impact on bed net effect for wide community protection either provided for free or under subsidized price [35,69,70]. In malaria-endemic parts of western Kenya and Ghana, studies have found that, the nets offered to pregnant mothers and infants during clinics visits do not mean utility for personal protection as many people own bed nets but do not use them [35,71].

## Conclusion

Distance to the health centre influenced malaria treatment seeking behaviors and the number of visits made by the household members. In addition, the education level of the household heads played a role in understanding and selecting malaria interventions for the household. Thus, locating health facilities or training and employing community health workers who will work with the endemic communities may improve malaria treatment seeking behavior, case management and hence reduce malaria associated morbidities, especially in high risk groups.

Education level of the household heads had a significant association with malaria treatment seeking behavior, bed net ownership and retreatment**.** The distance to the health facility significantly influenced the decision of the household heads to seek malaria treatment, especially for children. There is an urgent need for additional health officials and infrastructure in rural areas to be implemented by the Tanzania government.

## Competing interest

The authors have declared that no competing interests exist.

## Authors’ contributions

AL and EJK conceived and designed the study. AL, SM MJM, AMM, BJM, HDM, EEK performed the field work. AL, BJM, EEM and AMM analyzed the data: AL, EE, HDM and EJK wrote the paper. All authors reviewed and accepted the final version for submission.
